# Short report: interim safety results for a phase II trial measuring the integration of stereotactic ablative radiotherapy (SABR) plus surgery for early stage non-small cell lung cancer (MISSILE-NSCLC)

**DOI:** 10.1186/s13014-017-0770-7

**Published:** 2017-01-27

**Authors:** David A. Palma, Timothy K. Nguyen, Keith Kwan, Stewart Gaede, Mark Landis, Richard Malthaner, Dalilah Fortin, Alexander V. Louie, Eric Frechette, George B. Rodrigues, Brian Yaremko, Edward Yu, A. Rashid Dar, Ting-Yim Lee, Al Gratton, Andrew Warner, Aaron Ward, Richard Inculet

**Affiliations:** 1 Department of Radiation Oncology, London Health Sciences Centre, London, Canada; 2 Department of Oncology, Western University, London, Canada; 3 Department of Pathology, Western University, London, Canada; 4 Department of Medical Biophysics, Western University, London, Canada; 5 Department of Physics and Engineering, London Health Sciences Centre, London, Canada; 6 Department of Radiology, Western University, London, Canada; 7 Department of Surgery, Division of Thoracic Surgery, Western University, London, Canada; 8 Imaging Program, Lawson Health Research Institute, London, Canada

**Keywords:** Non-small cell, Lung cancer, Stereotactic ablative radiotherapy, Stereotactic body radiotherapy, Surgery

## Abstract

**Abstract:**

A phase II trial was launched to evaluate if neoadjuvant stereotactic ablative radiotherapy (SABR) before surgery improves oncologic outcomes in patients with stage I non-small cell lung cancer (NSCLC). We report a mandated interim safety analysis for the first 10 patients who completed protocol treatment. Operable patients with biopsy-proven T1-2 N0 NSCLC were eligible. SABR was delivered using a risk-adapted fractionation (54Gy/3 fractions, 55/5 or 60/8). Surgical resection was planned 10 weeks later at a high-volume center (>200 lung cancer resections annually). Patients were imaged with dynamic positron emission tomography-computed tomography scans using ^18^F-fludeoxyglucose (^18^F-FDG-PET CT) and dynamic contrast-enhanced CT before SABR and again before surgery. Toxicity was recorded using CTCAE version 4.0. Twelve patients were enrolled between 09/2014 and 09/2015. Two did not undergo surgery, due to patient or surgeon preference; neither patient has developed toxicity or recurrence. For the 10 patients completing both treatments, median age was 70 (range: 54–76), 60% had T1 disease, and 60% had adenocarcinoma. Median FEV_1_ was 73% predicted (range: 54–87%). Median time to surgery post-SABR was 10.1 weeks (range: 9.3–15.6 weeks). Surgery consisted of lobectomy (*n* = 8) or wedge resection (*n* = 2). Median follow-up post-SABR was 6.3 months. After combined treatment, the rate of acute grade 3–4 toxicity was 10%. There was no post-operative mortality at 90 days. The small sample size included herein precludes any definitive conclusions regarding overall toxicity rates until larger datasets are available. However, these data may inform others who are designing or conducting similar trials.

**Trial registration:**

NCT02136355. Registered 8 May 2014.

## Introduction

Stereotactic ablative radiotherapy (SABR, also called stereotactic body radiation therapy or SBRT) provides a promising therapeutic option for stage I non-small cell lung cancer (NSCLC) [1]. Many studies report 3-year local control rates of approximately 90%, based on post-treatment imaging [2]. However, the true pathologic complete response rate (pCR) is unknown. In most patients, local radiation-induced lung injury (RILI) is evident on CT after SABR and can impair the detection of local recurrences [3].

In operable patients undergoing surgery, neoadjuvant SABR could theoretically provide an oncologic benefit by decreasing the rate of positive margins, sterilizing the tumor to avoid the seeding of circulating tumor cells during surgery [4], or in some cases through an abscopal effect [5]. The MISSILE-NSCLC study was launched to evaluate the oncologic outcomes and toxicity after an *a priori* combined treatment approach of SABR followed by surgical resection, and to determine the pCR rate after SABR.

To our knowledge, the toxicity of this combined treatment approach has not been previously reported. Herein, we present the findings of an interim safety analysis.

## Methods

### Study design and patient selection

Institutional Research Ethics Board approval was obtained prior to study initiation, and the study was centrally registered (NCT02136355). Eligible patients had histologically-confirmed T1 or T2a (≤5 cm) NSCLC, with no evidence of nodal or distant metastases. Additional requirements included age ≥18, informed consent, Eastern Cooperative Oncology Group (ECOG) performance status 0–2, life expectancy > 6 months and a predicted post-operative forced expiratory volume in 1 second (FEV_1_) of ≥ 30%. Ineligibility criteria included patients with contraindication to either treatment, previous lung cancer within the past 5 years, previous thoracic radiation or a contrast allergy.

### Interventions

SABR was delivered according to a risk-adapted protocol, with the dose and fractionation dependent on the size and location of the tumor (54 Gy / 3 fractions, 55 Gy/5 fractions or 60 Gy/8 fractions). Treatment was delivered every second day regardless of the dose-fractionation regimen [6, 7]. A 4-D CT simulation scan was acquired for all patients. Respiratory gating was considered in cases where motion was > 7 mm in any direction.

The gross tumor volume (GTV) was defined as the visible tumor on CT imaging ± PET, and an internal GTV encompassed the GTV from all phases of respiration. No additional margin was included for microscopic spread of disease. A planning target volume (PTV) margin of 5 mm was used. The prescription point was approximately the 80% isodose line surrounding the PTV, with the requirement that 95% of the PTV was covered by 100% of the prescription dose.

Surgical resection was planned to occur at 10 ± 2 weeks following SABR and consisted of a lobectomy or sublobar resection by either an open or video-assisted thoracoscopic approach. All patients received sampling of high risk hilar and mediastinal lymph nodes at the time of resection. All procedures were done at a high-volume tertiary surgical centre, with a case volume of >200 lung resections annually.

Patients with pathologic node-positive disease (N1, N2, or N3) were referred for adjuvant chemotherapy. For patients with N2 or N3 disease, adjuvant radiotherapy to the mediastinum was to be considered as long as there was minimal overlap with the SABR dose distribution.

The trial protocol mandated that after 10 patients have been accrued and completed surgery, a safety analysis to review treatment toxicity would be undertaken separately by the study team and the data safety monitoring committee.

## Results

Twelve patients were enrolled between September 2014 and September 2015. Two did not undergo surgery following SABR due to concerns regarding medical operability: one continued to smoke more than two packs per day; the other had unrelated upper gastrointestinal dysfunction and possible gastroparesis resulting in poor functional status. Neither patient has developed toxicity or recurrence.

### Patient & treatment characteristics

For the 10 patients completing combined therapy, the median age was 70 (range: 54–76), 60% had T1 disease, and 60% had adenocarcinoma. Median FEV_1_ was 73% predicted (range: 54–87%). Median time to surgery post-SABR was 10.1 weeks (range: 9.3–15.6 weeks). Most patients underwent a lobectomy (*n* = 8) post-SABR, with the remainder receiving a wedge resection (*n* = 2). Median follow-up post-SABR was 6.3 months.

### Outcomes

A total of 24 toxicities were reported (Table 1). The most common toxicity was pain (grade 1–2), occurring in 90% of patients. Fatigue, pneumonia and pneumothorax each occurred in 20% of patients. There were 3 recorded grade 3–4 toxicities (pneumonia, atrial fibrillation and respiratory failure), all in the same patient, and all of which resolved. The highest grade of toxicity for each of the 10 patients is shown in Table 2. There were no deaths within 30 or 90 days of surgery.

**Table 1:** Type and grade of patient-reported toxicities

Toxicity	Number of Patients Reporting Toxicity by Grade^a^ (*N* = 24 total toxicities)					Total
	1	2	3	4	5	
Pain	2	7	0	0	0	9
Fatigue	2	0	0	0	0	2
Pneumonia	0	1	1	0	0	2
Pneumothorax	0	2	0	0	0	2
Atrial Fibrillation (uncontrolled)	0	0	0	1	0	1
Dyspnea	1	0	0	0	0	1
Respiratory failure	0	0	0	1	0	1
Post-Operative Bleeding	1	0	0	0	0	1
Broncho-pleural Fistula	0	1	0	0	0	1
Generalized Weakness	1	0	0	0	0	1
Nausea	0	1	0	0	0	1
Vomiting	0	1	0	0	0	1
Anorexia	1	0	0	0	0	1

^a^Note: Some patients developed >1 toxicity

**Table 2:** Highest grade of reported toxicity for each of 10 patients

Grade	Highest Toxicity Grade for Each Patient (*n* = 10)
	N (%)
1	1 (10%)
2	7 (70%)
3	0
4	1 (10%)
5	0

## Discussion

Neoadjuvant radiotherapy is part of standard treatment for certain cancers (e.g. rectal cancers, sarcomas), with the goal of reducing the risk of local recurrence or margin positivity. In stage I NSCLC, neoadjuvant ablative therapy has previously been explored using radiofrequency ablation (RFA) prior to surgical resection. However, the high prevalence of viable tumor cells after RFA (62% of cases) discouraged its widespread adoption [8]. Although prior studies have evaluated salvage surgery in selected patients who developed local recurrence after SABR [9–12], the use of a combined approach upfront has not been assessed previously.

While the sample size in this interim report is small, the toxicity rates reported herein compare favorably with those in prospective studies evaluating surgery alone. In a pooled analysis looking at two randomized studies comparing SABR versus lobectomy, the rate of grade 3–5 toxicity after lobectomy was 48% [13]. Toxicity rates appear to be lower after sublobar resection, often reported as approximately 30% [14]. However, comparisons across trials are subject to bias based on differences between study populations and treatments.

There is a strong relationship between hospital volume and surgical toxicity in NSCLC [15], and therefore the generalizability of these data to low-volume centers is unknown. In addition, the small sample size included herein precludes using the toxicity data to inform clinical practice until larger datasets are available. However, these data, compiled as a mandated interim safety report, are still informative. They sufficiently assure us it is safe to continue with combined treatment on the present clinical trial, which also provides reassurance to other groups who are designing or conducting similar trials. Furthermore, these promising early results generate valuable academic discussion, which garner interest in an understudied area and primes interested parties to the forthcoming results of the present trial. If there is a demonstrable benefit to combined treatment, there will be a need for validation studies to confirm a potential shift in practice patterns. If results are negative, other groups may reexamine the concept with alternative methodology. In either case, subsequent steps will require time. By inciting discussion now we hope to expedite our ability to answer important clinical questions that ultimately inform the best possible care for our patients.

## Conclusion

In conclusion, toxicity rates after combined SABR and surgical resection compare favorably with reported rates in prospective studies of surgical resection alone. While these findings are preliminary, and certainly inadequate to inform clinical practice, it provides an early assessment of the tolerability of combined treatment as we await mature data on pCR rates and oncologic outcomes.

## Abbreviations

18 F-FDG: 18 F-fludeoxyglucose
CT: computed tomography
CTCAE: Common terminology criteria for adverse events
ECOG: Eastern Cooperative Oncology Group
FEV_1_: Forced expiratory volume in 1 second
GTV: gross tumor volume
NSCLC: non-small cell lung cancer
pCR: pathologic complete response rate
PET: positron emission tomography
PTV: planning target volume
RFA: radiofrequency ablation
RILI: radiation-induced lung injury
SABR: stereotactic ablative radiotherapy
SBRT: stereotactic body radiation therapy
